# Effectiveness, working mechanisms, and implementation of youth-initiated mentoring for juvenile delinquents: a multiple-methods study protocol

**DOI:** 10.1186/s40352-024-00258-9

**Published:** 2024-02-15

**Authors:** Angelique Boering, Annabeth P. Groenman, Levi van Dam, Geertjan Overbeek

**Affiliations:** 1 Research Institute Child Development and Education, Preventive Youth Care Programme Group, University of Amsterdam, Nieuwe Achtergracht 127, 1018WS, Amsterdam, the Netherlands; 2 Dutch YIM Foundation, Amersfoort, the Netherlands; 3 Levvel Academic Centre for Child and Adolescent Psychiatry, Amsterdam, the Netherlands

**Keywords:** Youth-initiated mentoring, Informal mentoring, Juvenile delinquency, Selective prevention, Resilience

## Abstract

**Background:**

The societal costs associated with juvenile delinquency and reoffending are high, emphasising the need for effective prevention strategies. A promising approach is Youth-Initiated Mentoring (YIM). In YIM, professionals support youths in selecting a non-parental adult from within their social network as their mentor. However, until now, little (quasi-)experimental research has been conducted on YIM in the field of juvenile delinquency. We will examine the effectiveness, working mechanisms, and implementation of YIM as a selective prevention strategy for juvenile delinquents.

**Methods:**

This multiple-methods study consists of a quasi-experimental trial and a qualitative study. In the quasi-experimental trial, we aim to include 300 juvenile offenders referred to Halt, a Dutch juvenile justice system organisation which offers youths a diversion program. In the Netherlands, all juvenile offenders between 12 and 18 years old are referred to Halt, where they must complete the Halt intervention. Youths will be non-randomly assigned to region-matched non-YIM-trained and YIM-trained Halt professionals implementing Care as Usual (CAU, i.e., the Halt intervention) or CAU plus YIM, respectively. Despite non-random allocation, this approach may yield comparable conditions regarding (1) the characteristics of professionals delivering the intervention and (2) case type and severity. Youth and caregiver(s) self-report data will be collected at pre-and post-test and a 6-month follow-up and complemented with official Halt records data. Multilevel analyses will test whether youths following CAU plus YIM show a stronger increase in resilience factors and a stronger decline in the need for formal support and delinquency than youths following CAU. In the qualitative study, we will organise focus group interviews with YIM-trained professionals to explore boosters and barriers experienced by professionals during the implementation of YIM.

**Discussion:**

The proposed study will help identify the effectiveness of YIM in strengthening resilience factors and possibly decreasing juvenile delinquency. In addition, it may offer insights into how and for whom YIM works. Finally, this study can help strengthen the implementation of YIM in the future.

**Trial Registration:**

ClinicalTrials.Gov (# NCT05555472). Registered 7 September 2022. https://www.clinicaltrials.gov/ct2/show/NCT05555472?cond=Youth+Initiated+Mentoring&draw=2&rank=1.

## Introduction

Juvenile delinquency, defined as participating in unlawful behaviour as a minor or individual under statutory age, is highly prevalent during adolescence. In 2020, one-third of Dutch adolescents (36.6%) reported having committed at least one crime over the past year (Van der Laan & Beerthuizen, 2021), varying from vandalism to shoplifting to more severe forms of delinquency, such as fighting and threatening others with a weapon. Even though the development of adolescent delinquency follows an age-crime curve, with an upsurge and peak at 17 and a decline thereafter (Moffitt, 1993), about 34–45% of juvenile delinquents reoffends (Beerthuizen & Prop, 2021; Youth Justice Board, 2022). Moreover, the societal costs associated with juvenile delinquency and reoffending are high. Specifically, taking victim costs, criminal justice costs (e.g., police, legal aid, interventions), and loss of societal productivity into account, the costs associated with engaging in a lifelong criminal career may range from $1.7 to $5.3 million per person (e.g., Cohen, 1998; Cohen & Piquero, 2009; Koegl & Day, 2018), and the costs for reoffending by minors can go up to £1.5 billion (Newton et al., 2019). Over the past decades, the trend of “harsh” penal measures has strained governmental budgets while it has failed to reduce reoffending (Andrews & Bonta, 2010). Evidence suggests that early prevention efforts to avert criminal careers are more cost-effective than punitive measures (Welsh & Farrington, 2011, 2012). Consequently, it is crucial to intervene at an early stage of adolescents’ potential criminal careers and of utmost importance to develop interventions to prevent more persistent and violent patterns of delinquent behaviour.

Building effective interventions and prevention programs depends on identifying individual, familial and environmental risk factors linked to heightened delinquent behaviour (Farrington & Welsh, 2007; Nation et al., 2003). Several known key dynamic risk factors for reoffending, also known as *criminogenic needs*, are pro-criminal attitudes, aggressive behaviour, alcohol and drug use, education and employment, leisure and recreation, antisocial peers, and familial risk factors (e.g., Andrews & Bonta, 2010; Assink et al., 2015). Interventions targeting these criminogenic needs are believed to be most effective in reducing reoffending (Andrews & Bonta, 2010; Seigle et al., 2014). Thus, when turning to action-oriented prevention, one should primarily focus on strengthening factors that build resilience and, in turn, desistance against delinquent behaviour, such as increasing involvement with education, employment, leisure, or recreation and building positive relationships with family, peers and others within the social network (e.g., Fergus & Zimmerman, 2005; Seigle et al., 2014; Stagner & Lansing, 2009; Ward & Stewart, 2003; Zimmerman, 2013). Theoretically, Youth-Initiated Mentoring (YIM) is a promising approach for preventing reoffending because it directly or indirectly strengthens several of these factors (Van Dam & Schwartz, 2020; Schwartz et al., 2013).

## Youth-initiated mentoring

YIM is a novel and innovative approach to *natural* or *informal* mentoring (Van Dam & Schwartz, 2020; Schwartz et al., 2013). In contrast to *formal* mentoring programs, where youth are matched to non-parental adults who volunteer within a non-professional context, such as a school or community program (Raposa et al., 2019), *informal* mentoring programs focus on involving a non-parental adult from within the adolescents’ social network, someone with whom the relationship already *naturally* exists. In YIM, youth have the autonomy to choose their *natural* mentor and to *initiate* the mentoring relationship by asking the natural mentor to be their ‘YIM’. Professionals support this entire procedure of identifying, nominating, and officially positioning the YIM. The function of the YIM is twofold: being a confidant and ally for the youth and a partner to caregivers and professionals (Schwartz et al., 2013). In addition, the YIM is closely involved in shaping the support around the adolescents’ needs. Most importantly, the approach capitalises on the strengths and expertise of formal and informal support systems (Van Dam & Verhulst, 2016).

Strengthening natural mentor-mentee relationships alongside professional involvement has three main benefits over matched mentor-mentee relationships: it is more accessible, durable and empowers the social network. First, it is estimated that about 63–86% of youth can identify a natural mentor (e.g., Dang, 2012; Erickson et al., 2009; Hurd & Zimmerman, 2010; Tucker et al., 2019; Van Dam et al., 2017). This relatively large accessibility could counteract delays typically experienced in formal mentoring due to a larger need and interest in these programs than available volunteer mentors (Raposa et al., 2017; Spencer et al., 2019; Tucker et al., 2019). Second, evidence suggests that youth-initiated relationships are more durable than matched relationships, which increases the likelihood of positive youth outcomes (e.g., Schwartz et al., 2013; Grossman & Rhodes, 2002; Spencer et al., 2016). Approximately 74% of all informal mentors are still involved in the youths’ lives after two years (Van Dam et al., 2017), which could be explained by youths choosing their mentor, contributing to a better mentor-mentee match (Tucker et al., 2019). In contrast, matched mentors and mentees often struggle to form meaningful connections, causing the relationship to end prematurely (e.g., (Grossman et al., 2012; Grossman & Rhodes, 2002). Moreover, youth –particularly those in the juvenile justice system– may be more open to involving a mentor they know and trust (Spencer et al., 2019). Third, strengthening the social network empowers families. Parents who feel supported by their social network experience lower levels of parenting stress and engage in more positive parenting practices, such as monitoring and effective parenting (e.g., Ayala-Nunes et al., 2017; Ghazarian & Roche, 2010; McConnell et al., 2011; Taylor et al., 2015). Consequently, this and the youths’ perception of feeling supported might impact the youths’ behaviour, such as displaying less externalising and delinquent behaviour (e.g., Ghazarian & Roche, 2010; Hagen et al., 2005; Hatch et al., 2020; Wight et al., 2006). Thus, YIM has clear benefits over formal mentoring and has the potential to buffer against the development of juvenile delinquency.

## Youth-initiated mentoring for juvenile delinquents

YIM might be particularly well-suited for adolescents in the juvenile justice system (Spencer et al., 2019) because it relies heavily on three basic human needs that could be linked to the development of delinquent behaviour. Specifically, YIM has its theoretical underpinnings in the self-determination theory (Deci & Ryan, 2000), which holds that motivation to learn and develop occurs when an individual fulfils these three basic human needs: the need for autonomy, the need for relatedness, and the need for competence. These needs can be linked to several criminological theories on why youths engage in delinquent behaviour (Moffitt, 1993; Hirschi, 2017; Agnew, 2001, 2017). The overall assumption is that when these needs are met, delinquency in youth dissipates or decreases.

The need for autonomy implies that one feels the need to be a self-governing agent, the “desire to self-organize experience and behavior and to have activity be concordant with one’s integrated sense of self” (Deci & Ryan, 2000, p. 231). Both dual taxonomy theory (Moffitt, 1993) and strain theory (Agnew, 2001, 2017) argue that adolescents’ lack of perceived autonomy encourages delinquent behaviour. During adolescence, adolescents experience a mismatch between their desired and actual autonomy. The strain theory suggests that this ‘strain’ leads to frustration, leading individuals to cope with delinquent behaviour (Agnew, 2001, 2017). Empirical research supports the relationship between the need for autonomy and delinquency (e.g., Brezina, 2008; Van Petegem et al., 2015; Chen, 2010). YIM addresses this need for autonomy by letting the adolescent choose and ask their mentor themselves and working on self-concordant goals (i.e., goals aligned with one’s integrated sense of self) (Ruig & Van Dam, 2021).

The need for relatedness refers to how an individual experiences a sense of belonging and connections with important others (Deci & Ryan, 2000). The social control theory states that stronger bonds with society prevent individuals from engaging in delinquent behaviour (Hirschi, 2017). These bonds express themselves in four factors: (1) *attachment* (i.e., connections with significant others), (2) *involvement* (i.e., engagement in prosocial activities); (3) *commitment* (i.e., degree of connection to society), and (4) *beliefs* (i.e., the value someone places on the norms and values of the society). YIM enhances *attachment* and *commitment* by strengthening relationships with an important person from the social network. YIM might, therefore, impact youths’ degree of perceived social support and their perceived mattering by the active agreement and commitment of this significant other to be their mentor. YIM might also increase *involvement* because involvement in free-time activities, such as hobbies, organisations or clubs, and religious services, has been linked to an increased likelihood of having a natural mentor (Thompson & Greeson, 2017). The YIM could function as a role model for engaging in these prosocial activities as well as prosocial behaviours, but also in cultivating prosocial *beliefs.* Thus, YIM is likely to increase relatedness, which could lead to a decrease in delinquent behaviour.

The need for competence implies that an individual feels capable and effective in their behaviour (Deci & Ryan, 2000). The social learning theory of crime states that both conforming and deviant behaviour can be stimulated via social interactions, modelling, and reinforcement of the displayed behaviour (Akers, 2002). Specifically, the probability of engaging in delinquent behaviour increases when individuals associate with deviant or criminal individuals who either engage in criminal behaviour or have a pro-criminal attitude. Conversely, if one associates with individuals who display prosocial behaviour, one’s behaviour will likely shift in that direction (Akers, 2002). While more of an indirect effect, mentors might model and reinforce adolescents’ need for competence. The YIM could be a role model by providing guidance, sharing moral beliefs and skills, and reinforcing the youths’ self-esteem and prosocial behaviour. Research indicates that youths view their natural mentor as a “powerful role model” (Greeson et al., 2015; Spencer et al., 2016). In addition, YIM actively supports youths’ competence because youths are perceived and considered competent by professionals when choosing their mentors. Consequently, they feel competent to approach someone when help is needed in future situations (Van Dam & Verhulst, 2016; Ruig & Van Dam, 2021). To conclude, YIM might potentially, directly and indirectly, strengthen factors that build resilience and, in turn, desistance against delinquent behaviour.

## Effectiveness of youth-initiated mentoring

Even though YIM is relatively new and rigorous research on the approach has been limited, YIM has been identified as a promising strategy for the future of youth mentoring (Cavell et al., 2021). A recent scoping review on YIM identified nine peer-reviewed studies on the outcomes of YIM (Dantzer & Perry, 2022). YIM has been shown to impact positive youth outcomes, like improved psychological well-being, strengthened relationships, achievement of educational and occupational goals, and strengthened partnerships to support youth adjusting to independent living. These positive outcomes were observed in various vulnerable groups, such as youths who dropped out of high school, youths from multi-problem families receiving youth care, youths at risk for out-of-home-placement, and youths in foster care (e.g., Koper et al., 2021; Schwartz et al., 2013; Spencer et al., 2018; Van Dam et al., 2017). However, it should be noted that no strong conclusions can be drawn about the effectiveness of YIM, as seven out of nine studies adopted a qualitative study design, and none adopted a (quasi-)experimental study design. Moreover, the review only included studies explicitly using the term ‘Youth-Initiated Mentoring’. Noteworthy is that authors identified other studies on youth mentoring approaches that emphasise youths’ voice and choice of a mentor (e.g., C.A.R.E. or Connected Scholars) (Dantzer & Perry, 2022). A recent meta-analysis that included YIM and these similar approaches demonstrated that these approaches showed greater positive effect sizes on positive youth outcomes, such as academic functioning and social-emotional development, compared to formal mentoring approaches or the mere presence of a natural mentor (Van Dam et al., 2021).

Regarding delinquency, however, the findings of (similar) YIM approaches have been mixed. Some evidence suggests a decrease in the likelihood of conviction and engagement in delinquent activities (Millenky et al., 2010; Schwartz et al., 2013). At the same time, other studies indicate that these similar approaches have no impact on officially registered and self-reported delinquency nor delinquency-related outcomes, such as cognitive distortions, aggressive behaviour, substance use, and parenting behaviours (James et al., 2016; de Vries et al., 2017; De Vries et al., 2018). In conclusion, there is a need for (quasi-)experimental studies on the effectiveness of YIM in the field of juvenile delinquency. This offers the opportunity to determine whether YIM effectively or strengthens resilience factors and halts or reduces juvenile delinquency.

## The present study

Even though YIM seems particularly well-suited for use in a population of juvenile delinquents; currently, little is known about its effectiveness in the field of juvenile delinquency. Moreover, knowledge of mediators and moderators of potential YIM effects has yet to be documented. In addition, gaining insight into professionals’ experience implementing YIM is essential. In our multiple-methods multi-informant study, we will target these three main questions about the effectiveness (“Does YIM work?”), possible working mechanisms (“How does YIM work? And for whom does YIM work?”) and the implementation process (“What are boosters and barriers in the implementation of YIM?”). Our quasi-experimental trial will answer the first two questions, and our qualitative study will answer the last.

**Fig. 1:**
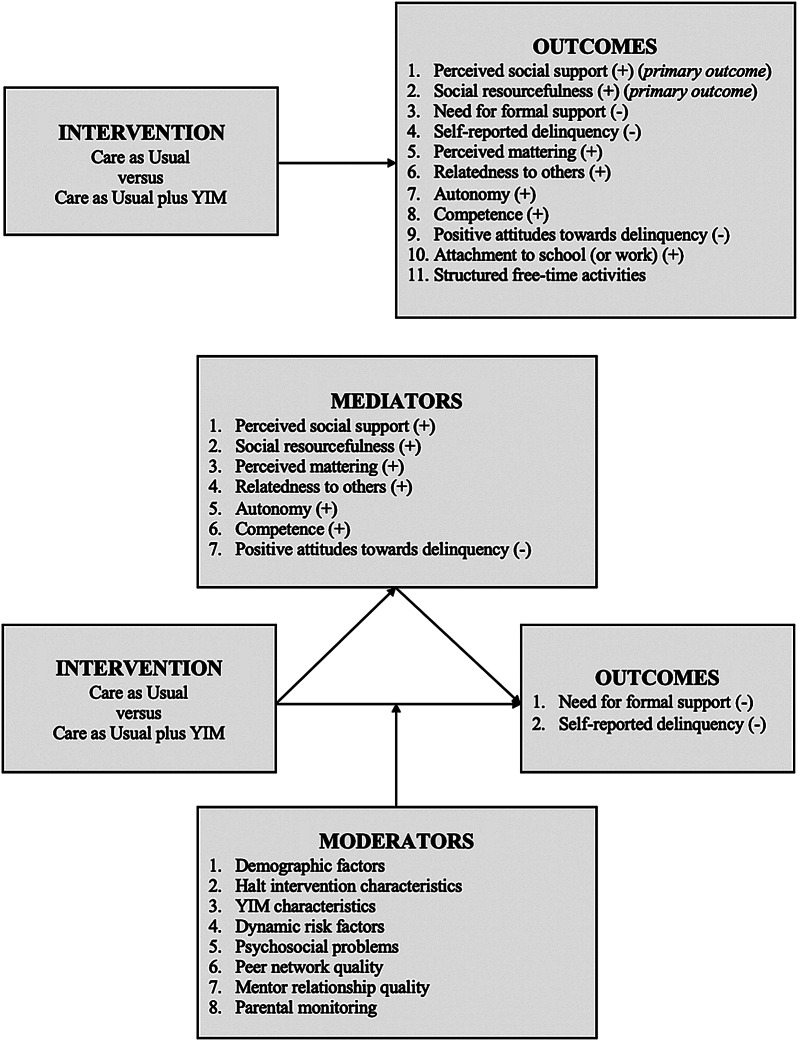
Theoretical model of YIM: outcomes, mediators, and moderators

Concerning effectiveness, we expect that YIM will strengthen youth resilience factors, such as perceived social support and social resourcefulness (*primary outcomes*), relatedness to others, autonomy, and competence. Moreover, we expect YIM to contribute to halting or decreasing the need for (formal) support and self-reported delinquency (*secondary outcomes*). Concerning the working mechanisms of YIM, we expect that an increase in resilience factors mediates the effect of halting or decreasing the need for (formal) support and self-reported delinquency. Furthermore, the impact of YIM will be larger for some individuals than for others, based on moderators such as demographic factors, psychosocial problems, mentor-mentee relationship quality (e.g., type of support, frequency of contact, relationship duration, (non)kin), peer network quality, parental monitoring, and treatment characteristics (i.e., Halt intervention characteristics and YIM characteristics). Also, see Fig. 1 for a pictorial depiction of the theoretical model. Finally, concerning the implementation of YIM, we will explore the boosters and barriers that facilitate or prevent its successful implementation. We intend to explore boosters and barriers within three domains: the innovation domain (i.e., characteristics of the innovation being implemented: YIM), the individuals’ domain (i.e., characteristics of individuals receiving the innovation: youth and caregivers, and characteristics of individuals delivering the innovation: Halt professionals) and the inner setting domain (i.e., characteristics of the setting in which the innovation is being implemented: Halt) (Damschroder et al., 2009, 2022; Fleuren et al., 2014).

## Methods and design

### Aims and setting

Our main aim is to examine whether YIM strengthens resilience factors, such as perceived social support and social resourcefulness, in delinquent youth. To do so, we will study YIM at Halt, a Dutch juvenile justice system organisation which offers youths a diversion program subsidised by the Ministry of Justice and Security. In the Netherlands, all juvenile offenders (12 to 18 years) are referred to Halt after committing relatively minor offences or crimes (Wolthuis & Stentoumi, 2023). Common offences and crimes include public intoxication, extreme school absenteeism, shoplifting and vandalism. Halt has the nationwide task of providing these youths with a tailored intervention aimed at preventing reoffending and increasing future opportunities (Halt, n.d.). Mainly, Halt works with first- and second-time offenders who remain in the community. Working with 15,000 youths yearly, Halt has a unique position to identify youths (and families) in vulnerable circumstances. These youths are guided towards the necessary formal support systems, such as youth care organisations or addiction treatment centres. Since 2019, 36 Halt professionals (15% of all Halt professionals) have been trained in YIM, allowing YIM-trained professionals to strengthen the informal support system by involving a non-parental adult from the youth’s social network. The Halt program is implemented throughout the Netherlands, but the organisation is organised into four central regions: (1) North-Holland-Centre, (2) North-East, (3) South, and (4) West. Each region has sub-teams supervised by a region manager, and within each region, multiple professionals have been trained in YIM. Of all YIM-trained professionals, 31 (86%) have agreed to collaborate with the YIM-Halt trial. More details on the content of the Halt intervention can be found under “Conditions” > “Care as Usual (CAU) condition”. The quasi-experimental trial (Study 1) and the qualitative study (Study 2) will be performed at Halt and are presented below.

### Study 1: quasi-experimental trial

#### Design

In this quasi-experimental design, youth referred to Halt are non-randomly allocated to either a non-YIM-trained Halt professional implementing *Care as Usual* (CAU; *N*_*professionals*_ = 31) or a YIM-trained Halt professional implementing *CAU plus YIM* (*N*_*professionals*_ = 31). Even though Halt’s distribution office allocates youth cases to professionals independent of case type and severity, they consider professionals’ workload within each region. This could lead to unequal distribution of youth cases over conditions. To ensure comparable conditions, the two groups of professionals are matched on educational level and region. Thus, despite non-random allocation, we expect similar conditions regarding (1) professionals’ characteristics and (2) case type and severity. Adolescents are our primary informants, but caregiver(s) of youths are also approached. This trial is registered at Clinicaltrials.gov (NCT05555472).

#### Co-creation

This study has been co-created with Halt professionals, policy officers, and management. They have been closely involved in decision-making concerning integrating the study into their regular working activities and giving their practical view on possible outcomes selected by the researchers based on previous research and the theory of YIM.

#### Study sample

**Fig. 2:**
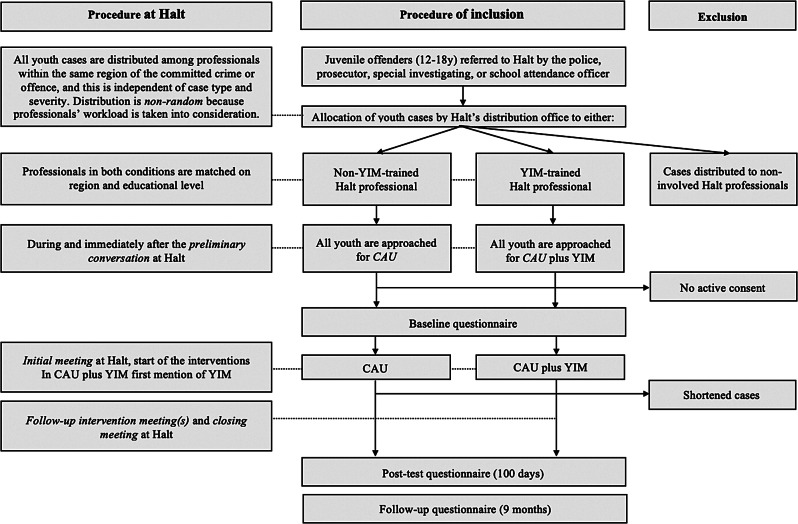
Participant flow through the study

Across both conditions, we aim to include 300 juvenile offenders who are (1) between 12 and 18 years old, (2) referred to Halt by either the police, a prosecutor, a special investigating officer, or a school attendance officer, and (3) required to follow a minimum of three meetings at Halt. Thus, so-called ‘shortened cases’, with only 1 or 2 meetings, will be excluded from participation beyond the first measurement (see Fig. 2).

#### Materials

Our primary outcome, with which we will assess the main intervention effect of YIM on perceived social support and social resourcefulness, will be measured with the Berlin Social Support Scales (BSSS), respectively the subscales ‘Perceived Social Support’ and ‘Support Seeking’ (Schwarzer & Schulz, 2003). All youth and caregiver(s)’ outcomes and measurement points are depicted in Table 1. In Table 2, an overview of measures administered to Halt professionals is presented.

**Table 1:** Overview of Youth and Caregiver(s) Measurements

Concepts		Measurement	Informants	# of items	Baseline	Post-test	Follow-up
Demographic factors		Demographics	Y	18–21	*		
Offense and referral information		Halt’s registration system	HP	-	*		
Halt intervention characteristics		Halt’s registration system	HP	-	*		
YIM characteristics		Halt’s registration system	HP	-		*	
Dynamic risk factors		Halt-SI	HP	-	*		
Psychosocial problems		Halt-SI SDQ	HP Y	- 20	* *		
Prosocial behaviour		SDQ	Y	5	*	*	*
**RESILIENCE FACTORS**:							
**1**	**Quality social network**:						
	1.1 Perceived social support	BSSS, *perceived social support*	Y, C	8	*	*	*
	1.2 Perceived mattering	GMS	Y	5	*	*	*
	1.3 Relatedness to others	BPNS, *relatedness to others*	Y	4	*	*	*
	1.4 Peer network quality	IDF	Y	8	*	*	*
	1.5 Mentor relationship quality	Items relational process YsoR	Y, C Y, C	11 6		*	*
**2**	**Social skills**:						
	2.1 Social resourcefulness	BSSS, *support seeking*	Y, C	5	*	*	*
**3**	**Feelings of autonomy and competence**:						
	3.1 Competence satisfaction	BPNS, *competence*	Y	4	*	*	*
	3.2 Autonomy satisfaction	BPNS, *autonomy*	Y	4	*	*	*
**4**	**Attitudes towards delinquency**:		Y		*	*	*
	4.1 Cognitive biases	HIT-Q, *cognitive biases subscales*	Y	39	*	*	*
**5**	**Parental monitoring and family outcomes**						
	5.1 Adolescent disclosure	PMS, *AD*	Y, C	6	*	*	*
	5.2 Parental solicitation 5.3 Family resilience 5.4 Parental stress	PMS, *PS* FES PSS	Y, C C C	6 14 18	* * *	* * *	* * *
**6**	**Commitment to social environment**:						
	6.1 Attachment to school	SCS, *value of school* NSCR, *school form*	Y	11	*	*	*
	6.2 Structured free-time activities	IFTA and TSWF	Y	4–8	*	*	*
**NEED FOR FORMAL SUPPORT**		BSSS, *need for support* IHR	Y, C	8	*	*	*
**REOFFENDING**							
	Self-reported delinquency Youth delinquent behaviour	AHSRD CBCL, *delinquent behaviour*	Y C	17 13	*	*	*

*Note Informants*. *HP* Halt professionals; *C* Caregiver(s); *Y* Youths

*Note Questionnaires. AD* Adolescent Disclosure; *AHSRD* Add Health Self-Reported Delinquency; *BPNS* Basic Psychological Needs Scale; *BSSS* Berlin Social Support Scale; *CBCL* Child Behaviour Check List; *FES* Family Empowerment Scales; *GMS* General Mattering Scale; *Halt-SI* Risk Assessment Tool Halt; *HIT-Q* How I Think Questionnaire; *IDF* Items Delinquent Friends (Van der Laan & Blom, 2006); *IFTA* and *TSWF * Items Free-Time Activities and Time Spent With Friends (Van der Laan & Blom, 2006); *IHR *Items Halt Research (Ferwerda, 2006); *PMS* Parental Monitoring Scales; *PPI* Peer Pressure Inventory; *PS* Parental Solicitation; *PSS* Parental Stress Scale; *NSCR* Netherlands Institute for the Study of Crime and Law Enforcement; *SCS* School Connectedness Scale; *SDQ* Strength and Difficulties Questionnaire; *WODC* Research and Documentation Centre in the Netherlands; *YsoR* Youth Strength of Relationship; *YSR* Youth Self Report

**Table 2:** Overview of Halt Professional Measurements

Concepts	Measurement	Informant	# of items	Before recruitment
Demographic factors	Questionnaire	HP	4	*
Educational level	Questionnaire	HP	4	*
Work experience	Questionnaire	HP	2	*
Outcome Expectancy	OES	HP	3	*
Personality	QBF	HP	30	*

*Note Informants. HP* Halt professionals

*Note Questionnaires. OES* Outcome Expectancy Scales; *QBF* Quick Big Five

#### Recruitment

Non-YIM-trained Halt professionals will recruit participants for the CAU condition, and YIM-trained professionals will recruit participants for the CAU plus YIM condition. Professionals in both conditions explicitly invite minors (and their caregivers) to participate in research to improve the Halt intervention. This is done verbally during the preliminary conversation when the youth is first registered at Halt and by sending a flyer with core information about the study immediately after this preliminary conversation. A QR-code or link on this flyer refers youth (and their caregivers) to an online consent form (categories: 12–15 years, 16 + years, caregivers). Participants can voluntarily sign up for the study via this route.

#### Conditions

**Care as Usual (CAU) condition.** Participants (*N* = 150) will follow the regular Halt intervention implemented by non-YIM-trained Halt professionals, also referred to as *Care as Usual.* Aside from inviting youth to participate, professionals are instructed to follow their regular working activities. During the initial meeting, the content and duration of the Halt intervention (in hours and the number of meetings) are determined by a compulsory screening and risk assessment (i.e., Halt-SI) and the nature of the offence. Tailored to the youths’ needs, the professional then selects activities from five different modules: (1) reflection on one’s behaviour, (2) parental involvement, (3) social skills training, (4) victim-offender reconciliation, and (5) future opportunities. For example, activities in the module ‘social skills training’ are ‘modeling’ and ‘dealing with peer pressure’. It is important to note that victim-offender reconciliation is compulsory for all youth (e.g., writing an apology letter to the victim or providing restitution). After selecting activities, the professional draws up an official contract with agreements on the course of the intervention—signed by the youth. The youth then proceeds to fulfil the activities selected by the professional. Some activities can be done at home, such as writing an apology letter, while others are practised during an intervention meeting with a professional, such as social skills training. Importantly, professionals discuss the completion of each activity with the youth. During the closing meeting, the intervention is completed positively if the youth has complied with all agreements made at the start of the trajectory (Wolthuis & Stentoumi, 2023). If deemed necessary, youth are referred to formal support. The aim is to complete CAU within 100 days. The number of meetings with the professional can vary from one to six, with a minimum intensity of 1 h and a maximum intensity of 20 h. On average, youths follow three meetings: an initial meeting, an intervention meeting, and a closing meeting. The initial meeting is in person, while other meetings can be in person or online. Eighty-five per cent of caregivers are present during the initial and closing meetings and provide support where necessary.

**CAU plus YIM condition.** Participants (*N* = 150) will follow the regular Halt intervention described above in combination with YIM, which can be selected as an activity from the module ‘future opportunities’. If selected, it replaces other Halt activities. During the initial meeting of Halt, professionals in this condition help all youth to identify a mentor by asking the YIM question (i.e., “Who in your environment (an adult, aside from your caregiver(s)) can you turn to if necessary?”). If at least three meetings are necessary, professionals explain what YIM entails and support youth by using the network or YIM assignment, which helps to gain insight into the youth’s network. A positive or negative reaction of the youth can follow. A minimum of four (online) meetings are integrated into Halt’s regular working activities if the youth has identified and nominated a YIM. The first meeting is a conversation between the Halt professional and the YIM. They discuss the reaction of the YIM to being asked by the youth, the help needed by the youth, the YIM’s expectations for the trajectory and the role of the professional. The professional explains that the mentor and the youth have the autonomy to form the trajectory to the youth’s needs and the mentor’s possibilities. The mentor will function as a confidant and ally to the youth in various situations (e.g., listening to the youth or supporting the youth with schoolwork). Mentors are not trained because, in YIM, it is believed that the YIM has all the knowledge and tools to support the youth. The professional has a facilitating role, meaning that they initiate the mentoring trajectory and, if necessary, can be reached for support on the YIM’s initiative. The second meeting officially positions the “YIM”. The youth, caregivers, YIM, and professional discuss each other’s expectations, formulate goals, agree on the support frequency, and plan the evaluation meeting. Thus, the type and frequency of support may vary over youth cases. The mentor can partner with caregiver(s) and professionals by making agreements regarding specific goals (e.g., ensuring a youth goes to school in the case of extreme school absenteeism). Again, the professional takes a facilitating and supportive role. In the third meeting, the professional and the YIM have interim contact to discuss the trajectory and if they can proceed with the evaluation meeting. If this is not the case, they plan another interim contact moment. However, if this is the case, the fourth meeting is a joint evaluative meeting during which the youth, caregivers, YIM, and professional evaluate the goals and agreements made at the start of the trajectory. They also discuss how to proceed and whether it is possible to end Halt’s involvement. If so, this also functions as a closing meeting. Similar to CAU, the completion of Halt is evaluated by the Halt professional, while the completion of YIM is evaluated by all parties (i.e., youth, caregiver(s), YIM and professional). Moreover, the aim is to complete CAU plus YIM within 100 days. Importantly, because YIM meetings are integrated into Halt’s regular working procedures and replace other Halt activities, we expect the same number of meetings between the youth and the professional. However, extra meetings between the youth and their YIM are not uncommon, and the YIM often remains involved after Halt’s involvement has ended.

#### Data collection and procedure

Both recruitment and data collection started on September 1st, 2022. Recruitment will stop after 15 months because researchers strive to complete pre- and post-tests within 18 months. Follow-up measurements can occur up to 24 months after the start of the data collection. Participants will be asked to fill out three questionnaires: before the initial meeting (baseline), 100 days after baseline (post-test), and nine months after baseline (follow-up). After giving consent via the flyer, participants will automatically be referred to the first online questionnaire and instructed to complete it before their initial meeting at Halt. After that, researchers will approach eligible participants for post-test and follow-up measurements (see Fig. 2). All questionnaires will be administered in Dutch and will take approximately 30 min to complete. Participants will receive €7,50 per completed questionnaire; when they complete all questionnaires, they may win AirPods (€130).

Complementary to the questionnaires, we will receive pseudonymised youth case data from Halt’s registration system. Professionals in both conditions will register the characteristics of the Halt intervention (e.g., number of meetings, type of crime) and the risk assessment (i.e., Halt-SI) per youth case. In the CAU plus YIM condition, professionals will additionally register characteristics of YIM (e.g., the reaction of youth to YIM, caregivers’ approval of YIM, and the youth’s relation to the YIM (e.g., uncle, grandmother)). Most importantly, professionals will report the steps they have taken to implement YIM: (1) the YIM question was asked (“Who, an adult aside from your caregiver(s), can you turn to if necessary?”), (2) steps were taken to support youth in identifying and nominating a YIM, such as the network or YIM assignment, and (3) a YIM was positioned in an official meeting with all parties. Note that the third level of implementation is only possible if youths can identify and nominate a mentor.

#### Data analyses

First, for both the effect analyses on the different outcome variables and moderator and mediator analyses, we will perform multiple regression analyses with baseline levels of the outcome variable as a covariate and condition as a predictor variable. Second, to correct for the clustering of participants within regions and within Halt professionals, the regression analyses will be performed in a multilevel model with the participant as level 1, the professional as level 2, and the region as level 3 cluster variables. Third, to correct for multiple testing, we will implement a Benjamini Hochberg False Detection Rate (FDR; (Benjamini & Hochberg, 1995) correction on the p-values for the respective outcome tests. Fourth, to test for the similarity between conditions, we will compare the two groups of Halt professionals and both conditions on several characteristics (e.g., demographic factors, professionals’ educational level, or youths’ psychological problems). If differences are detected between conditions, we will use propensity score matching (PSM) techniques (Caliendo & Kopeinig, 2008) to match participants in the CAU plus YIM condition to participants in the CAU condition. Lastly, we will implement the Intention-to-Treat (ITT) principle for handling missing data on post-test and follow-up measurements (Detry & Lewis, 2014; Yeatts & Martin, 2015). Thus, we will analyse all missing data of participants according to their assigned condition, regardless of whether the intervention was received or completed (Detry & Lewis, 2014; Yeatts & Martin, 2015). Missing data points will be estimated by performing Full Information Maximum Likelihood (FIML) methods. We will consider the percentage of missing data and patterns of missing data (e.g., missing completely at random) (Bennett, 2001; Schulz & Grimes, 2002).

**Power Analysis.** To assess the sample size per condition for the primary and secondary outcomes, we performed an a priori power analysis in G*Power 3.1 (Faul et al., 2009). To attain a power level of 0.80 with a significance level of α = 0.05, a total sample size of *N* = 100 is necessary to detect a small effect size (*f* = 0.10) in a linear multiple regression analysis. Meta-analyses that explored the effects of formal mentoring programs on delinquent behaviour and reoffending revealed small overall effects, both *d* = 0.21 (~ f = 0.10) (Jolliffe & Farrington, 2007; Tolan et al., 2014). For moderator and mediator analysis, a larger sample size is required. We performed a priori power analysis a Monte Carlo Power Analysis (Schoemann et al., 2017, p. 384). For a simple trivariate correlation, a total sample size of *N* = 150 is necessary to attain a power level of 0.80 with a significance level of α = 0.05. This would indicate *N* = 75 per condition. However, we aim to include *N* = 150 per condition because (1) we will also include time as a factor in the moderator and mediation models, and (2) we expect relatively high drop-out rates of over 20% (e.g., drop-out at post-test or follow-up measurements) (Furlan et al., 2009).

### Study 2: qualitative study

#### Design

This study aims to gain insight into the implementation process of YIM. To do so, we will conduct focus group interviews with YIM-trained Halt professionals during regularly scheduled supervision meetings. We will first conduct a large focus group, followed by three smaller focus groups. The large focus group aims to gain an in-depth understanding of the implementation of YIM in a specific youth case, while the small focus groups aim to gain insight into boosters and barriers in the implementation process of YIM at Halt. If necessary, we will conduct additional focus groups or individual interviews (i.e., significant variations exist between focus groups).

#### Study sample, recruitment, and data collection

At Halt, 36 professionals are trained in YIM, of which 31 will recruit data for our trial. We will approach YIM-trained Halt professionals during regularly scheduled supervision meetings. All YIM-trained Halt professionals who want to participate in the study will participate in the large focus group, followed by a smaller focus group consisting of 6 to 9 participants (Finch et al., 2014). If individual interviews or additional focus groups need to be conducted, researchers will adopt similar recruitment strategies or approach Halt professionals for individual interviews.

#### Materials

We will develop a discussion format for both types of focus groups (Finch et al., 2014). Based on observations and literature, the format will contain questions within three domains of the Consolidated Framework of Implementation Research (CFIR): the innovation domain (i.e., characteristics of the innovation being implemented: YIM), the individuals’ domain (i.e., characteristics of individuals receiving the innovation: youth and caregivers, and characteristics of individuals delivering the innovation: Halt professionals) and the inner setting domain (i.e., characteristics of the setting in which the innovation is being implemented: Halt) (Damschroder et al., 2009, 2022; Fleuren et al., 2014). For example, “What aspects do you find easy (or difficult) in the implementation of YIM?” or “How do you experience the organisational support to implement YIM?”.

#### Procedure

Professionals will be informed about the qualitative study and must consent if they want to participate. Each participant will follow the large focus group, followed by a smaller one. In the large focus group, we will recreate a *reflecting team* model (Van Dam, 2007; Chang, 2010): in the inner circle, participants will engage in structured discussion about a Halt professional’s prior youth case (*N* = 6–9), and in the outer circle, four smaller circles (*N* = 3–5) will observe the discussion through a specific perspective: (1) the youth; (2) caregivers or YIMs; (3) Halt professional; or (4) Halt. Afterwards, the different perspectives will be discussed in plenary, and all participants will be encouraged to share their thoughts and perspectives. In the smaller focus groups, structured discussions with Halt professionals will be activated via *think-pair-share* techniques (Lyman, 1981). First, participants will be instructed to *think* about the posed question; second, they will be asked to discuss this question in *pairs* and to write down their answers; and third, the pairs will be asked to *share* their findings in the group, after which a discussion will be initiated. This technique encourages individual participation, makes discussions more productive, and improves the quality of the responses (Kagan, 1994). The researchers will collect the *pair* forms, and participants will be thanked and asked whether they have any additional thoughts to share.

#### Data analyses

Focus groups (and possible individual interviews) will be recorded, and these audio files will be transcribed. To identify patterns of themes in the data, we will follow a multi-step thematic analysis approach (Braun & Clarke, 2006). We will perform the six steps of the approach: familiarise with the data, generate initial codes, search for themes, review the themes, define and name the themes, and report on the identified themes. We will use inductive coding strategies for the initial codes, after which we will perform deductive coding strategies based on the CFIR (Damschroder et al., 2009, 2022).

## Discussion

With this study on YIM, a new examination is initiated in the field of youth mentoring for delinquent behaviour. Research on YIM remains limited; thus, we aim to contribute to the further understanding of the effectiveness of YIM as a selective prevention strategy for juvenile delinquency. To do so, we will perform a quasi-experimental trial simultaneously targeting youth resilience and delinquency outcomes and a qualitative study in which professionals will evaluate their experiences with implementing YIM. Moreover, examining how and for whom the YIM works may help to inform a tailored approach when introducing YIM in the field of juvenile delinquency.

The current study has three main strengths. First, we will examine the effectiveness and not the efficacy of YIM. The interventions (i.e., CAU and CAU plus YIM) are implemented under real-life circumstances, allowing us to study the performance of YIM in daily practice rather than the performance under ideal and controlled circumstances (Singal et al., 2014). Thus, the study has high ecological validity (Andrade, 2018). Second, the study is designed in co-creation with Halt professionals, policy officers and management. Real-life circumstances were the starting point of this co-created study: “How can we integrate the study into professionals’ regular working activities?”. Joint-decision making has contributed to the seamless integration of the study into professionals’ working activities and increased their involvement in and support for the study. Third, the study will focus on both resilience and delinquency outcomes. Even though we will examine whether YIM is effective as a selective prevention strategy for juvenile delinquency, we will also focus on youth resilience factors in delinquent youth. By including outcomes relevant to positive youth development, we consider adolescents’ developmental trajectories. By strengthening the social network, YIM might build resilience (Fergus & Zimmerman, 2005; Seigle et al., 2014; Stagner & Lansing, 2009; Ward & Stewart, 2003; Zimmerman, 2013) and, in turn, desistance against juvenile delinquency. Therefore, it is crucial to include these measurements pertinent to youth development.

It is also crucial to mention three potential challenges. First, allocating youths to either condition (i.e., to a non-YIM-trained or YIM-trained professional) is non-random. Random allocation is impossible because Halt’s distribution office considers professionals’ workload. This might lead to an unequal division of youth cases over conditions and, thus, less comparable conditions. Differences between conditions could then possibly be explained by confounding factors instead of the effects of YIM, such as differences in the severity of the youth case (Flannelly et al., 2018). To ensure comparable conditions, we will match professionals in the CAU plus YIM condition within each region to professionals in the CAU condition. Nevertheless, we must be cautious of drawing causal inferences. We will also address other confounding factors between conditions, such as differences in the number of sessions or types of activities. Second, baseline and post-test measurements will occur without the knowledge of the implementation level of YIM in the CAU plus YIM condition. We will assess three levels of implementation steps taken by a professional: (1) the YIM question was asked, (2) steps were taken to support youth in identifying and nominating a YIM, and (3) a YIM was positioned in an official meeting. External factors can influence these steps, such as a youth not being able to identify or nominate a YIM. If this is the case, it is impossible to position a YIM. We will, however, instruct professionals to explain and motivate YIM in all youth cases, possibly leading to the positioning of a YIM. Overall, we expect that all cases will at least have the second level of implementation. However, due to the natural course of YIM, we cannot rule out natural variation in the level of implementation. Because this might impact the findings, we will consider these implementation levels in our analyses. Moreover, these implementation levels will allow us to explore whether different elements of YIM affect the approach’s effectiveness. Third, all participants will voluntarily sign up for the study, potentially leading to a selective pool of more intrinsically motivated participants. Even though Halt has a broad population, with varying degrees of problems and different types and severity of offences and crimes, the registered group might not entirely represent the Halt population. Potentially, youth with more severe problems might be missed. This would, however, apply to both conditions – if this were to be the case. Hence, to appeal to the entire population at Halt, we will offer a monetary contribution per completed questionnaire and the chance to win the grand prize, namely AirPods. Fourth, a challenge in the focus group interviews is that being in a group, people might not feel comfortable enough to share their true perspectives and opinions on working with the innovation. Moreover, some individuals could be more dominant in the conversation than others, which might discourage some individuals from actively participating. To encourage active participation and a comfortable environment, we will perform *think-pair-share* techniques (Kagan, 1994; Lyman, 1981) in a neutral environment without the involvement of YIM and Halt management.

Halt’s population consists of 15,000 juvenile minors each year. Including a representative population of Halt allows for studying how and for whom YIM might work. This knowledge would benefit (Halt) professionals in decision-making on how and when to implement YIM. At the same time, youths who benefit from the approach can be specifically targeted, leading to a tailored approach. Suppose YIM is an effective addition to Halt’s short-term intervention. In that case, YIM can be further implemented within their intervention and potentially more widely within the juvenile justice system, such as juvenile detention centres, even outside the Netherlands. Insight into factors that facilitate or prevent YIM implementation yields specific strategies that impact these boosters or negate these barriers. These challenges and strategies can be considered and adopted by others who aim to implement YIM or a new intervention embedded within an organisation. Overall, this study will contribute to knowledge on whether YIM is a suitable informal mentoring approach for youth within the juvenile delinquency field. Moreover, information on which program elements of YIM work can further strengthen informal mentoring approaches.

This study will investigate the effectiveness of YIM for juvenile delinquents. Subsequently, this study will be able to answer questions considering the working mechanisms and possible boosters and barriers experienced in implementation processes. In conclusion, we will contribute to a further understanding of informal mentoring approaches as a selective prevention strategy within the juvenile delinquency field.

## Data Availability

Halt organises access to the sample; data will be collected by the author(s). All authors have access to the datasets. After signing a confidentiality agreement, research assistants or students working on the project have access to pseudonymised data. The datasets generated and/or analysed during the current study are not publicly available due to sensitivity and confidentiality. Still, they are available from the corresponding author on a reasonable request via a secured access repository after completing the study.

## Abbreviations

CAU: Care as Usual
CAU plus YIM: Care as Usual plus Youth-Initiated Mentoring
FDR: False Detection Rate
ITT: Intention-to-Treat
PSM: Propensity Score Matching
QR-Code: Quick Response Code
YIM: Youth-Initiated Mentor *or* Youth-Initiated Mentoring
